# Lurasidone in the long-term treatment of Japanese patients with bipolar I disorder: a 52 week open label study

**DOI:** 10.1186/s40345-021-00230-8

**Published:** 2021-08-02

**Authors:** Teruhiko Higuchi, Tadafumi Kato, Mari Miyajima, Kei Watabe, Takahiro Masuda, Katsuhiko Hagi, Jun Ishigooka

**Affiliations:** 1 Japan Depression Center, Tokyo, Japan; 2 The National Center of Neurology and Psychiatry, Kodaira, Japan; 3 Laboratory for Molecular Dynamics of Mental Disorders, RIKEN Center for Brain Science, Wako, Saitama Japan; 4 Department of Psychiatry, Juntendo University, Tokyo, Japan; 5 Sumitomo Dainippon Pharma Co., Ltd, 13-1, Kyobashi 1-Chome, Chuo-ku, Tokyo, 104-8356 Japan; 6 Institute of CNS Pharmacology, Tokyo, Japan

**Keywords:** Lurasidone, Bipolar disorder, Atypical antipsychotic

## Abstract

**Background:**

The current study evaluated the long-term (52 week) safety and impact on symptom measures of lurasidone (with or without lithium or valproate) for the treatment of bipolar I disorder in Japanese patients.

**Methods:**

Bipolar patients for this open-label flexibly dosed lurasidone (20–120 mg/day) study were recruited from those with a recent/current depressive episode who completed an initial 6 week, double-blind, placebo-controlled, lurasidone study (depressed group), and those with a recent/current manic, hypomanic, or mixed episode (non-depressed group) who agreed to enroll directly into the long-term study. Measures of adverse events and safety included treatment-emergent adverse events, vital signs, body weight, ECG, laboratory tests, and measures of suicidality and extrapyramidal symptoms. Symptom measures included Montgomery Åsberg Depression Rating Scale (MADRS) and Young Mania Rating Scale (YMRS).

**Results:**

The most common adverse events associated with lurasidone were akathisia (30.7%), nasopharyngitis (26.6%), nausea (12.1%), and somnolence (12.1%). Minimal changes in lipids and measures of glycemic control occurred. Mean change in body weight was + 1.0 kg in the non-depressed group and − 0.8 kg in the depressed group. MADRS total scores declined by a mean (SD) of 2.0 (14.7) points from long-term baseline to endpoint in the depressed group who had received placebo in the prior 6 week trial. The depressed group that had received lurasidone during the prior 6 week study maintained their depressive symptom improvements. For the non-depressed group, YMRS total scores decreased over time.

**Limitations:**

No control group was included, treatment was open-label, and 49.7% of patients completed the 52 week study.

**Conclusions:**

Long-term treatment with lurasidone 20–120 mg/day for Japanese patients with bipolar disorder maintained improvements in depressive symptoms for depressed patients who were treated in a prior 6 week trial and led to improvements in manic symptoms among a newly recruited subgroup of patients with a recent/current manic, hypomanic, or mixed episode. Few changes in weight or metabolic parameters were evident.

*Clinical trial registration:* JapicCTI-132319, clinicaltrials.gov—NCT01986114.

**Supplementary Information:**

The online version contains supplementary material available at 10.1186/s40345-021-00230-8.

## Introduction

Bipolar disorder is a chronic and disabling disorder that affects both social and occupational functioning to an extensive degree (Dean et al. 2004; Fajutrao et al. 2009; Gardner et al. 2006; Vos et al. 2012). The lifetime prevalence of bipolar disorder worldwide is about 1.0% (bipolar I disorder: 0.6%; bipolar II disorder: 0.4%), with an additional 1.4% experiencing a subthreshold version of the disorder (Merikangas et al. 2011). Bipolar disorder is associated with the loss of more disability-adjusted life years than Alzheimer’s disease and all forms of cancer (World Health Organization 2002). Though the majority of patients with bipolar disorder experience recurrent depressed episodes more commonly than manic or hypomanic episodes (Calabrese et al. 2004; Judd et al. 2002), about half of those with mania experience severe role impairment (Merkiangas et al. 2011). Those individuals with bipolar I disorder have been found to be symptomatic on average approximately 70% of the time (Forte et al. 2015).

In Japan, clinical guidelines have recommended the use of lithium, sodium valproate (VPA), and several atypical antipsychotic agents in the treatment of mania episodes associated with bipolar disorder (Kanba et al. 2013). In the treatment of bipolar depression, three medications (quetiapine, olanzapine, and lurasidone) have received approval in Japan. Because of the chronic nature of bipolar disorder, long-term safety and effectiveness data is critical for ongoing management of recurring episodes. The long-term management of bipolar disorder takes on further significance given the high rates of metabolic syndrome and cardiovascular disease associated with this disorder (de Jong et al. 2018; Vancampfort et al. 2013) and the association of metabolic syndrome with adverse clinical outcomes in those with bipolar disorder (Bai et al. 2016). Given the fact that medical conditions related to metabolic syndrome and obesity emerge over time, and the episodic nature of bipolar depression, it is important to evaluate safety and symptom changes for medications for bipolar depression over the course of long-term treatment. Only a few studies of one year or more have investigated safety and symptom changes of agents for bipolar disorder in a Japanese population (e.g., Kanba et al. 2019; Katagiri et al. 2014).

The atypical antipsychotic drug lurasidone has high affinity for D2, 5-HT7 and 5-HT2A receptors (antagonist), moderate affinity for 5-HT1A receptor (partial agonist), and no appreciable affinity for H1 and M1 receptors (Ishibashi et al. 2010). The efficacy of lurasidone as a monotherapy and as an adjunctive therapy with lithium or VPA has been established for the acute treatment of bipolar depression (Loebel et al. 2014a, b; Kato et al. 2020). Two longer terms studies of lurasidone for bipolar depression have been conducted. In one extension study, 24 weeks of open-label lurasidone treatment was well tolerated with minimal effects on weight and metabolic parameters and continued improvements in depressive symptoms (Ketter et al. 2016). A second 28 week open-label extension study of lurasidone monotherapy also found minimal changes in body weight and metabolic parameters and further improvements in depressive symptoms (Ishigooka et al. 2021). In the first study (Ketter et al. 2016), 16.6% of the sample had an Asian background. In the second study, 28.3% of patients were Japanese (Ishigooka et al. 2021).

Despite the three short-term positive trials of lurasidone as a monotherapy or adjunctive therapy for bipolar depression (Loebel et al. 2014a, b; Kato et al. 2020), and the two extension trials of 24 and 28 weeks (Ketter et al. 2016; Ishigooka et al. 2021), the generalizability of the results to a Japanese population and to bipolar patients with a most recent manic, hypomanic, or mixed episode is uncertain. In addition, safety and symptom changes over the course of longer term (beyond 28 weeks) lurasidone treatment for bipolar disorder has not been reported. The aims of the current study were to provide data on safety and symptom changes over the course of one year of open-label lurasidone treatment for bipolar disorder, including those with a most recent depressed, manic, hypomanic, or mixed episode, among Japanese patients.

## Methods

### Patients

The study population consisted of two groups of Japanese patients. In the first group were Japanese patients who had completed an initial 6 week, double-blind, placebo-controlled, evaluation (Kato et al. 2020) of lurasidone in the treatment of bipolar depression and enrolled in a subsequent 52 week open-label extension phase. This group is labeled the “depressed episode” sample here. In the second group were Japanese patients with bipolar I disorder whose current or most recent episode was a mania (ie, meeting criteria for DSM-IV-TR specifier 296.4x), hypomania (DSM-IV-TR specifier 296.40), or mixed (DSM-IV specifier 296.6×) episode. This group is labelled the “non-depressed episode” sample here. This latter group of patients enrolled directly into a 52 week long-term lurasidone study (without a preliminary 6 week double-blind phase).

Patients from both routes of enrollment were male and female outpatients 18–74 years of age. For the non-depressed group, patients who were willing to initiate treatment with lithium or VPA adjunctively were enrolled. The 6 week, double-blind study enrolled patients with a DSM-IV-TR diagnosis of bipolar I disorder (with or without rapid cycling) who were currently experiencing a major depressive episode of at least 4 weeks but less than 12 months in duration. Patients who had completed the 6 week double-blind trial and were considered to be without safety concerns were eligible for the subsequent long-term study. For the second route of enrolment, patients with a DSM-IV-TR diagnosis of bipolar I disorder (with or without rapid cycling) were eligible if their current or most recent episode was a mania, hypomania, or mixed episode. For both the group of patients who had participated in the prior 6 week trial and the newly recruited patients who did not have an initial 6 week double-blind phase, the key exclusion criteria were: (1) presence of another Axis I or Axis II disorder other than bipolar disorder that was the focus of treatment received in the 3 months prior to screening, (2) a score of ≥ 4 on MADRS item 10 (suicidal thoughts) (at week 6 for those who participated in the prior 6 week double-blind study; at screening or baseline for those newly recruited patients who did not have an initial 6 week double-blind phase), (3) had a history of nonresponse to an adequate (6 week) trial of three or more antidepressants, antipsychotics, lithium, or VPA during the current episode, and (4) were judged to be an imminent risk of suicide, injury to self, others, or property. Diagnosis was determined using the Mini-International Neuropsychiatric Interview (Sheehan et al. 1998).

The 6 week double-blind study, the subsequent long-term extension phase, and the study recruiting patients with a manic, hypomanic, or mixed episode directly into a 52 week long-term treatment period, were each reviewed and approved by institutional review boards at each investigational site. The studies were conducted in accordance with the International Conference on Harmonization Good Clinical Practices guidelines and with the ethical principles of the Declaration of Helsinki. All patients reviewed and signed an informed consent document explaining study procedures and potential risks prior to enrollment in the 52 week long-term treatment study.

### Study design

This study was a 52 week open-label flexible-dose study of lurasidone with or without a lithium or VPA. The initial 6 week double-blind study recruited patients at 102 centers in 8 countries. Only those recruited at 76 centers in Japan were included in the current report. The study, including the double-blind treatment phase and 52 week open-label phase, was conducted between January 22, 2014 and February 17, 2018.

All patients who had participated in the prior 6 week double-blind study started the 52 week extension study at a lurasidone dose of 60 mg/day for 1 week to maintain the blind of the 6 week trial until the 6 week dataset was ready for analysis. For the newly recruited patients who did not participate in an initial 6 week double-blind study, the starting dose was 20 mg/day followed by a gradual dose escalation. Lurasidone was taken once daily, within 30 min after the evening meal and administered at a flexible dose (20–120 mg/day) for 52 weeks. The dose of lurasidone could be increased or decreased by 20 mg/day at the scheduled visits, and could be increased by 20 mg/day at unscheduled visits at least 7 days after the prior visit. Dose increases were only permitted once a week between scheduled visits. When any safety concerns were raised, the dose could be reduced by 20 mg/day at unscheduled visits without waiting for 7 days.

### Concomitant medications

Concomitant use of either lithium or VPA was not mandatory for the completers of the prior 6 week double-blind study because these drugs were prohibited in that prior study. The decision to add a mood stabilizer for patients who had completed the prior 6 week study was a clinical decision by the investigator at each site based on patient symptoms. For patients who had not participated in the prior study (non-depressed episode sample), concomitant use of either lithium or VPA was mandatory in order to evaluate the long-term efficacy and safety of lurasidone adjunctive to a mood stabilizer. The dose of lithium and VPA could be modified based on the patient’s condition and serum concentrations that were obtained at each study visit. If necessary, the investigator could use either lithium or VPA at a lower or higher dose than approved with prior consultation with the medical monitor. If any safety concerns were raised, treatment with either lithium or VPA was to be terminated.

Prohibited during the 6 week double-blind and the 52 week long-term study were CYP3A4 inhibitors and inducers, Chinese herbal medications, Ginkgo Biloba extract, Kava Kava, depot antipsychotics, MAO inhibitors, clozapine, fluoxetine, olanzapine/fluoxetine combination, electroconvulsive therapy, and St. John’s wort. Lorazepam (≤ 2 mg/day) was permitted as needed (but prohibited within 8 h of an assessment) for the treatment of anxiety, agitation, irritation, and other psychiatric symptoms/signs. Hypnotics were permitted for insomnia. For patients untreated with antiparkinson agents at screening, such medications were prohibited from screening until the initiation of the study treatment. For patients who had completed the prior 6 week study and who had received antiparkinson drugs or other drugs for extrapyramidal symptoms at the initiation of the study treatment, the drugs were to be continued without any dosage modification. For patients who had not participated in the prior study and who had received antiparkinson drugs or other drugs for the treatment of extrapyramidal symptoms at screening, drugs for extrapyramidal symptoms were titrated down and terminated before initiating study treatment. If any extrapyramidal symptoms developed or worsened after the initiation of the study treatment, permitted antiparkinson medications included biperiden (≤ 16 mg), trihexyphenidyl (≤ 10 mg), benztropine (≤ 6 mg), and diphenhydramine (≤ 300 mg). For akathisia, propranolol (≤ 120 mg/day), amantadine (≤ 300 mg/day), or one of the allowable antiparkinson medications was permitted.

### Safety and tolerability assessments

Safety and tolerability were assessed throughout the study by the incidence and severity of treatment-emergent adverse events (TEAEs), laboratory measures of prolactin, glucose metabolism, and lipid metabolism, vital signs, body weight, and QTc interval determined from electrocardiography (ECG) measurements. Treatment-emergent mania was defined in depressed group as a YMRS total score of ≥ 16 at any 2 consecutive post-long-term-baseline visits or at the final assessment, or an adverse event of mania or hypomania. The influence of treatment on extrapyramidal symptoms was evaluated by the occurrence of extrapyramidal AEs, the proportion of patients using concomitant antiparkinson drugs, changes on the Drug-Induced Extrapyramidal Symptoms Scale (DIEPSS; Inada et al. 2003) total score (excluding overall severity). The proportion of patients with any instance of suicide attempt or suicidal ideation was measured by the Columbia-Suicide Severity Rating Scale (C-SSRS; Chappell et al. 2012). The DIEPSS, C-SSRS, body weight, and vital signs were obtained at long-term study baseline and post-long-term baseline weeks 1, 2, 4, and every 4 weeks up to week 52. ECGs and laboratory tests were obtained at long-term baseline and week 4 and every 8 weeks up to week 52.

### Symptom and functioning assessments

Symptom and functioning measures included the Montgomery-Åsberg Depression Rating Scale (MADRS; Montgomery and Åsberg 1979) total score (range: 0–60), Young Mania Rating Scale (YMRS; Young et al. 1978) (range: 0–60), the Clinical Global Impression: Bipolar Version -Severity of Illness (CGI-BP-S; Spearing et al. 1997) depression score (range: 1–7), the Sheehan Disability Scale (SDS; Sheehan 1983) total score (range: 0–30), and the Hamilton Anxiety Rating Scale (HAM-A; Hamilton 1959) (range: 0–56). Trained raters conducted the assessments at each visit. The MADRS, YMRS, and CGI-BP-S were obtained at long-term baseline and post-long-term baseline weeks 1, 2, 4, and every 4 weeks up to week 52. The SDS and HAM-A were obtained at long-term baseline and week 4 and every 8 weeks up to week 52.

An additional endpoint was the proportion of patients who experienced a recurrence or relapse of a mood event from clinical stability. Recurrence/relapse of a mood event was defined as the occurrence of any of the following: (1) fulfilled DSM-IV-TR criteria for major depressive, manic, hypomanic, or mixed episode; (2) required treatment intervention for depressive, manic, hypomanic, or mixed symptoms with antipsychotics (other than the study drug), antidepressants, mood stabilizers (other than lithium and VPA), anxiolytics, or benzodiazepines (treatment with lorazepam, temazepam, zolpidem, zolpidem CR, eszopiclone, or zaleplon within the permitted dose range was not applied to this criterion), (3) psychiatric hospitalization for any bipolar mood episode, (4) YMRS or MADRS total score ≥ 18 or at least one of the CGI-BP-S (overall, depression, mania) scores ≥ 4 at 2 consecutive visits no more than 10 days apart, or (5) discontinuation from the study because of a mood event (as determined by the investigator). Clinical stability (sustained response) of bipolar disorder was defined as total scores of ≤ 12 on the MADRS and YMRS over at least 12 weeks, with the allowance of 2 exceptions (the YMRS and/or MADRS total scores up to 13 or 14, respectively) as long as these exceptions did not occur during the last 4 weeks before achieving clinical stability. Time to recurrence/relapse following the achievement of clinical stability was also analyzed, as was time until all-cause discontinuation.

### Statistical analysis

All analyses were performed on the population of patients who took at least one dose of long-term study drug. Week 6 of the double-blind study was considered to be the baseline of the long-term study for patients who were enrolled in the prior study. Descriptive statistics were used to summarize safety and symptom/functioning data. Number and percent of patients with TEAEs over the course of the 52 weeks were summarized. Mean (SD) change from long-term baseline to week 52 was calculated for laboratory tests, vital signs, body weight, ECG parameters, and the DIEPSS. Symptom and functioning variables were examined in terms of mean changes from long-term baseline to endpoint (LOCF). For time to clinical stability and time to all cause discontinuation, median and its 95% confidence interval using the product-limit method were calculated. The numbers and percentages of subjects who experienced a recurrence/relapse and clinical stability were summarized. Analyses were conducted for all patients, and also separately for patients who received lurasidone and for those who received placebo in the prior 6 week double-blind study, as well as for the patients who had not participated in the prior study as a group and separately by type of current/recent episode.

## Results

### Patients and disposition

**Fig. 1:**
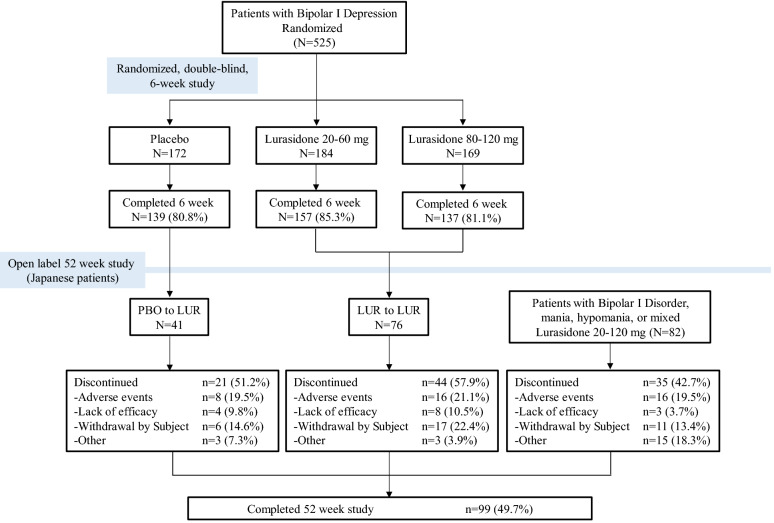
Study flow diagram and subject disposition during 52 weeks of long-term treatment with lurasidone. Depressed episode group consisted of Japanese patients who had completed an initial 6 week, double-blind, placebo-controlled, evaluation of lurasidone. PBO to LUR = received placebo during prior 6 week trial followed by lurasidone (flexibly dosed 2–120 mg/day) in 52 week long-term trial. LUR to LUR = received lurasidone (flexibly dosed at either 20–60 or 80–120 mg/day) during prior 6 week trial followed by lurasidone (flexibly dosed 20 to 120 mg/day) in 52 week long-term trial. Non-depressed episode group consisted of Japanese patients with bipolar I disorder whose current or most recent episode was a mania, hypomania, or mixed episode. *LUR* lurasidone, *PBO* placebo

Figure 1 presents the full flow of patients starting with the 6 week double-blind, placebo-controlled trial and the additional recruitment of patients with bipolar I disorder who had a most recent episode of mania, hypomania, or mixed features and did not participate in the prior 6 week trial. Enrolled in the 52 week long-term study were 41 Japanese patients who received placebo and 76 who received lurasidone during the 6 week double-blind study, plus an additional 82 Japanese patients who did not participate in the prior 6 week trial, for a total sample of 199. All of these patients received at least one dose of study drug and were included in the safety population for analyses. Of the Japanese patients who completed the 6 week trial, only 7 did not enroll in the 52 week trial. The demographic and clinical characteristics of these 7 patients were comparable to the characteristics of the patients who did enroll in the 52 week trial. Of the Japanese patients enrolled in the 52 week long-term study who received placebo during the initial 6 week double-blind trial, 51.2% (n = 21) discontinued the long-term study prior to 52 weeks. Of the Japanese patients who received lurasidone during the initial 6 week trial, 57.9% (n = 44) discontinued prior to the end of the 52 week long-term phase. Of the patients who did not participate in the 6 week double-blind trial, 42.7% (n = 35) discontinued prior to the end of the 52 week long-term study. Overall, 20.1% (n = 40) of those enrolled in the 52 week long-term study discontinued because of adverse events; 7.5% (n = 15) discontinued due to lack of efficacy and 17.1% (n = 34) chose to withdraw from the study.

The full group of patients enrolled in the 52 week long-term study were on average 41.6 years of age with approximately an equal percent of men and women (Table 1). Among the patients in the non-depressed episode group, the most recent episode was manic for 43 patients, hypomanic for 27 patients, and mixed for 12 patients. As expected, the average level of depressive symptoms, as measured by the MADRS and CGI-BP-S depression score was higher for those patients who participated in the prior double-blind study that required a current episode of depression at double-blind baseline, compared to the patients who did not participate in the prior double-blind trial and who had a most recent manic, hypomanic, or mixed bipolar episode (for the MADRS, Cohen’s d effect size using the larger SD of the two groups = 0.85; for the CGI-BP-S, d = 0.96). Similarly, the mean YMRS score was higher for non-depressed episode group compared to the depressed episode group (d = 0.78). The long-term baseline demographic and clinical characteristics of patients who had received placebo in the prior 6 week trial were similar to those that had received lurasidone (Additional file 1: Table S1).

**Table 1:** Baseline characteristics of patients treated in 52 week long-term study

	Most recent or current episode of bipolar I disorder		
	All Patients (n = 199)	Depressed episode (n = 117)	Non-Depressed episode (n = 82)
Demographics and clinical characteristics			
Male, n (%)	102 (51.3)	63 (53.8)	39 (47.6)
Age years, mean (SD)	41.6 (12.0)	40.0 (10.9)	43.8 (13.1)
Duration of bipolar I disorder, mean years (SD)	13.5 (11.1)	13.8 (10.6)	13.0 (11.7)
With rapid cycling, n (%)	30 (15.1)	15 (12.8)	15 (18.3)
Efficacy measure at long-term study baseline			
MADRS, mean (SD)	12.4 (10.3)	16.0 (10.2)	7.3 (8.0)
CGI-BP-S Overall, mean (SD)	2.98 (1.29)	3.10 (1.25)	2.80 (1.33)
CGI-BP-S Depression, mean (SD)	2.51 (1.39)	3.03 (1.31)	1.77 (1.13)
CGI-BP-S Mania, mean (SD)	1.77 (1.18)	1.23 (0.56)	2.55 (1.39)
SDS, mean (SD)#	11.9 (8.7)	13.7 (9.03)	9.2 (7.6)
YMRS, mean (SD)	4.7 (6.7)	2.0 (2.8)	8.7 (8.6)
HAM-A, mean (SD)	8.3 (7.2)	10.6 (7.5)	5.0 (5.2)
Safety measure at long-term study baseline			
DIEPSS (excluding overall severity), mean (SD)	0.36 (0.78)	0.50 (0.87)	0.16 (0.60)

^#^Number of patients: All = 181, Depressed = 109, Non-Depressed = 72

*CGI-BP-S* Clinical Global Impression Bipolar Version Severity of illness score, *DIEPSS* Drug-Induced Extrapyramidal Symptoms Scale, *HAM-A* Hamilton Rating Scale for Anxiety total score, *LUR* lurasidone, *MADRS* Montgomery-Åsberg Depression Rating Scale, *PBO* placebo, *SD* Standard Deviation, *SDS* Sheehan Disability Scale, *YMRS* Young Mania Rating Scale

At long-term baseline, 53.7% of the non-depressed episode sample had been receiving a mood stabilizer. Of the 44 patients in the non-depressed episode group who were already taking a mood stabilizer at long-term baseline, 23 (52.3%) were receiving lithium, 19 (43.2%) were receiving VPA, and 2 (4.5%) were receiving both lithium and VPA. As per protocol, all of the remaining patients in the non-depressed episode sample received a mood stabilizer going forward from the long-term study baseline.

### Study drug exposure

The mean (SD) daily dose of lurasidone for all patients (n = 199) during the 52 week study was 57.0 (26.1) mg and the modal dose was 57.0 mg/day. The number (percent) of patients who received a modal daily dose for each possible lurasidone dosage was: 20 mg: 50 (25.1%), 40 mg: 37 (18.6%), 60 mg: 54 (27.1%), 80 mg: 24 (12.1%), 100 mg: 19 (9.5%), and 120 mg: 15 (7.5%). In the overall safety population, the mean (minimum, maximum) percent compliance (number of tablets taken / number of tablets should have been taken) × 100) throughout the study was 98.9%. The mean (SD, median) number of days of drug exposure for the 199 participants was 233.8 (143.3, 343).

### Safety

Overall, 84.9% (n = 169) of patients in the 52 week study experienced at least one treatment-emergent adverse events (Table 2). The most common adverse events were akathisia (30.7%), nasopharyngitis (26.6%), nausea (12.1%), and somnolence (12.1%). Rates of adverse events were generally similar for the depressed episode group compared to the non-depressed episode group. Rates of TEAEs were also generally similar for patients who had received placebo vs lurasidone during the prior 6 week trial and also for type of recent or current episode among patients in the non-depressed patients (Additional file 1: Table S2).

**Table 2:** Treatment-emergent adverse events (safety population; *N* [%]; incidence ≥ 5%)

Adverse event	Most recent or current episode of bipolar I disorder		
	All patients (n = 199)	Depressed episode (n = 117)	Non-Depressed episode (n = 82)
At least one adverse event	169 (84.9%)	96 (82.1)	73 (89.0%)
Akathisia	61 (30.7%)	40 (34.2%)	21 (25.6%)
Nasopharyngitis	53 (26.6%)	24 (20.5%)	29 (35.4%)
Nausea	24 (12.1%)	12 (10.3%)	12 (14.6%)
Somnolence	24 (12.1%)	10 (8.5%)	14 (17.1%)
Weight increased	17 (8.5%)	8 (6.8%)	9 (11.0%)
Headache	16 (8.0%)	7 (6.0%)	9 (11.0%)
Parkinsonism	15 (7.5%)	10 (8.5%)	5 (6.1%)
Disease progression	13 (6.5%)	9 (7.7%)	4 (4.9%)
Vomiting	13 (6.5%)	8 (6.8%)	5 (6.1%)
Diarrhoea	10 (5.0%)	4 (3.4%)	6 (7.3%)
Dystonia	10 (5.0%)	5 (4.3%)	5 (6.1%)

The incidence of TEAEs did not increase with duration. The most common TEAEs leading to discontinuation were disease progression (n = 10), followed by akathisia (n = 8), and insomnia, mania, suicidal ideation, and suicide attempt (n = 2 each).

For the full sample, TEAEs related to extrapyramidal symptoms were reported for 17.6% (35 of 199) of patients up to week 52. None of these TEAEs were serious and all were mild or moderate in severity. Treatment-emergent metabolic AEs were reported for 13.1% (26 of 199) of patients across the long-term treatment period. Among these, glucose urine present and diabetes mellitus in 1 patient each were recorded as serious. Mood-event related TEAEs were reported for 13.1% (26 of 199) patients up to week 52. Among these, disease progression in 3 patients, mania and suicide attempt in 1 patient each were serious TEAEs. Treatment-emergent mania occurred in 0.9% (1 of 117) patients of the depressed group. Other significant adverse events are presented in Additional file 1: Table S3, separately for placebo vs lurasidone for those enrolled in the prior 6 week trial and separately for type of most recent/current episode for those non-enrolled in the prior 6- week trial. Suicidality-related TEAEs up to week 52 included suicide attempt in 2 patients and suicidal ideation in 4 patients. No patients had a completed suicide and no other deaths occurred in the study. A weight increase of ≥ 7% occurred for 17.7% (n = 35) of patients; a weight decrease of ≥ 7% occurred for 14.5% (n = 29) of patients.

An increase in the mean HOMA-IR (fasting) from long-term study baseline to week 52 was evident (mean change = 0.37) for the full sample, with similar mean changes in both the depressed episode and non-depressed episode groups (Table 3). The mean concentration of triglycerides (fasting) increased from long-term study baseline to week 52 (mean change = 5.4 mg/dl). This change was primarily driven by a mean change of 10.0 mg/dl for the depressed group (enrolled from the prior 6 week trial) compared to a mean change of 0.3 mg/dl in the non-depressed group (who did not participate in the prior trial). Except for the above, no clinically relevant changes from long-term baseline were noted in clinical chemistry, hematology, urinalysis, endocrine parameters, or vital signs. No meaningful differences in laboratory parameters or body weight were evident for those who received placebo vs. lurasidone in the prior 6 week trial, or depending on type of recent/current episode within the non-depressed sample (Additional file 1: Table S4). Overall mean change in body weight to week 52 was 0 kg (-0.8 kg in the depressed group; + 1.0 kg in the non-depressed group). Mean changes from long-term baseline to week 52 in ECG parameters were not clinically meaningful. No subject had a post-long-term baseline QTcF interval of > 500 ms.

**Table 3:** Body weight and laboratory parameters: mean (SD) change from long-term baseline to week 52 (observed case analysis)

Parameter	Most recent or current episode of bipolar I disorder		
	All	Depressed episode	Non-Depressed episode
Body Weight, kg Mean change Median change	n = 99 0.0 (5.6) 0.1	n = 52 − 0.8 (5.6) 0.1	n = 47 (5.5) 0.1
Triglycerides (mmol/L) -fasting Mean change Median change	n = 99 0.061 (0.667) 0.068	n = 52 0.113 (0.734) 0.073	n = 47 0.003 (0.587) 0.057
Total cholesterol (mmol/L) -fasting Mean change Median change	n = 99 − 0.080 (0.656) − 0.026	n = 52 − 0.065 (0.695) − 0.091	n = 47 − 0.098 (0.617) 0.052
LDL cholesterol (mmol/L) -fasting Mean change Median change	n = 97 − 0.039 (0.545) 0.026	n = 50 − 0.064 (0.603) 0.039	n = 47 − 0.012 (0.482) 0.026
HDL cholesterol (mmol/L) -fasting Mean change Median change	n = 99 − 0.047 (0.276) − 0.052	n = 52 − 0.018 (0.255) − 0.039	n = 47 − 0.079 (0.297) − 0.052
Blood Glucose (mmol/L) -fasting Mean change Median change	n = 99 0.044 (0.606) 0.111	n = 52 0.045 (0.690) 0.111	n = 47 0.044 (0.504) 0.056
Insulin (pmol/L) -fasting	n = 98 9.68 (63.54) 9.72	n = 51 8.42 (76.05) 9.03	n = 47 11.05 (47.14) 10.42
HOMA-IR -fasting Mean change Median change	n = 98 0.37 (2.76) 0.33	n = 51 0.34 (3.46) 0.30	n = 47 0.40 (1.73) 0.35
Hemoglobin A1C (%) Mean change Median change	n = 99 0.00 (0.29) 0.00	n = 52 − 0.01 (0.35) 0.00	n = 47 0.01 (0.20) 0.00
Prolactin, (μg/L), Overall Mean change Median change	n = 98 − 1.7 (17.6) − 0.5	n = 51 − 5.5 (18.7) − 1.3	n = 47 2.3 (15.5) 1.3
Prolactin, (μg/L), Male Mean change Median change	n = 44 − 0.3 (9.2) − 0.3	n = 24 0.3 (7.5) − 0.5	n = 20 − 0.9 (11.1) 0.3
Prolactin, (μg/L), Female Mean change Median change	n = 54 − 3.0 (22.2) − 0.6	n = 27 − 10.6 (23.8) − 2.6	n = 27 4.7 (17.9) 3.7

No notable changes (LOCF) from long-term baseline were found in the DIEPSS total score at week 52 (mean change = 0.0). Based on the results of C-SSRS, any suicidality after long-term baseline was reported for 26.3% (52 of 198) of patients in the full sample.

The proportion of patients in the full sample who received one or more concomitant medications during the 52 week treatment period was 94.5%. There were 38.2% who received antiparkinson medications, 57.3% mood stabilizers, 41.2% anxiolytics, and 67.3% hypnotics. When the results were summarized by the most recent or current episode of bipolar I disorder (depressed vs. non-depressed), the proportion of patients using concomitant medications was similar in the depressed episode group (90.6%) and the non-depressed episode group (100%). Antiparkinson medications were more commonly used in patients with a depressive episode (47.9% for depressed, 24.4% for non-depressed; the same order applies hereafter), while mood stabilizers (27.4%, 100%) and hypnotics (59.8, 78.0%) were more commonly used in patients with a non-depressed episode. The proportions of patients with anxiolytics were relatively similar in the two groups (37.6, 46.3%).

### Changes in symptoms and functioning

Table 4 summarizes the effect of up to 52 weeks of treatment with lurasidone on measures of affective symptoms and functioning based on a conservative LOCF-endpoint analysis. For both subgroups of depressed patients (double-blind placebo and double-blind lurasidone) minimal mean changes were noted during up to 52 weeks of treatment in depressive symptom measures (MADRS or CGI-BP-S depression), in mania symptom measures (YMRS or CGI-BP-S mania), and in the SDS functional measure. Similarly, for the group with a non-depressed episode, minimal mean changes were noted during up to 52 weeks of treatment in both depressive and mania symptom measures, and the SDS functional measure. On an observed case analysis (Additional file 1: Figures S1 and S2), improvement in the mean MADRS was noted during up to 52 weeks of treatment with lurasidone in both subgroups of depressed patients (double-blind placebo and double-blind lurasidone).

**Table 4:** Mean change (SD) from long-term baseline to LOCF endpoint in efficacy measures

Measure	Most recent or current episode of bipolar I disorder		
	Depressed episode		Non-Depressed episode (n = 81)
	PBO to LUR (n = 41)	LUR to LUR (n = 76)	
	Mean (SD)	Mean (SD)	Mean (SD)
MADRS	− 2.0 (14.7)	1.4 (12.9)	2.5 (10.9)
YMRS	− 0.6 (3.4)	0.2 (3.6)	− 4.8 (9.0)
CGI-BP-S overall	− 0.39 (1.60)	0.12 (1.43)	− 0.14 (1.66)
CGI-BP-S depression	− 0.32 (1.64)	0.13 (1.58)	0.38 (1.53)
CGI-BP-S mania	− 0.07 (0.52)	0.03 (0.73)	− 0.86 (1.43)
HAM-A†	− 1.1 (8.5)	− 0.3 (8.9)	0.9 (6.2)
SDS#	− 0.4 (10.7)	0.8 (9.7)	− 0.9 (9.9)

^†^Number of patients: All = 197, PBO to LUR = 41, LUR to LUR = 75, Non-Depressed = 81

^#^Number of patients: All = 177, PBO to LUR = 38, LUR to LUR = 69, Non-Depressed = 70

PBO to LUR = received placebo during prior 6 week trial followed by lurasidone (flexibly dosed 20 to 120 mg/day) in 52 week long-term trial

LUR to LUR = received lurasidone (flexibly dosed at either 20–60 or 80–120 mg/day) during prior 6 week trial followed by lurasidone (flexibly dosed 20–120 mg/day) in 52 week long-term trial

*CGI-BP-S* Clinical Global Impression Bipolar Version Severity of illness score, *HAM-A* Hamilton Rating Scale for Anxiety total score, *LUR* lurasidone, *MADRS* Montgomery-Åsberg Depression Rating Scale, *PBO* placebo, *SD* Standard Deviation, *SDS* Sheehan Disability Scale, *YMRS* Young Mania Rating Scale

Changes in symptom and functioning measures based on type of recent/current episode for the non-depressed episode group are shown in Additional file 1: Table S5. On the YMRS, substantial improvement over the first 12 weeks was seen for the non-depressed episode group, regardless of type or presence of concomitant mood stabilizer (Additional file 1: Figures S3 and S4).

The proportions of patients with all-cause discontinuation were similar across the all treatment groups continuing from the prior study and newly recruited subjects. The median time to all-cause discontinuation was 343 days. Based on the Kaplan–Meier analysis, the probability of all-cause discontinuation at week 52 was 50.3%.

Of the 199 enrolled patients, 103 (51.8%) achieved clinical stability over the course of the 52 week treatment period (Additional file 1: Table S6). The mean duration of treatment until achieving stabilization was 141.3 ± 79.94 days. The proportion of subjects achieving clinical stability was 44.4% (52 of 117) in patients with a depressed episode (51.2% for those who had receive placebo 40.8% for those who had received lurasidone in the prior 6 week trial), and 62.2% (51 of 82) in patients with a most recent non-depressed episode. Within the non-depressed group, clinical stability was achieved those with a most recent manic, hypomanic, and mixed episode by 69.8%, 41.7%, and 59.3%, respectively. The mean duration of treatment until achieving stabilization was 160.6 ± 92.41 days for the depressed episode group and 121.7 ± 59.58 days for the non-depressed episode group. The proportion of subjects with recurrence/relapse of any mood event up to Week 52 was 12.5% (6 of 48) in patients with a depressed episode and 23.5% (12 of 51) in patients with a non-depressed episode. The median time to recurrence/relapse could not calculated.

## Discussion

This 52 week long-term study found no new safety concerns over the course of long-term treatment with 20–120 mg/day of flexibly-dosed lurasidone with or without a mood stabilizer within a sample of Japanese patients with bipolar disorder. There were no notable differences in the incidence of TEAEs and common TEAEs between patients with a most recent/current depressed episode and those with a most recent/current non-depressed episode. The incidence of TEAEs did not increase with duration of treatment. No patients in the current study had a post-long-term baseline QTcF interval of > 500 ms and there were no completed suicides. Overall, the results of the current study suggest that 52 weeks of treatment with lurasidone, in doses up to 120 mg/day, has a relatively low potential for causing adverse weight and metabolic effects. As such, the current study extends the safety findings of previous studies of lurasidone (Loebel et al. 2014a; Kato et al. 2020; Ketter et al. 2016) for bipolar depression to a Japanese population and to bipolar patients with a most recent manic, hypomanic, or mixed episode. Importantly, the current safety findings also extend the shorter-term findings of previous studies of lurasidone for bipolar disorder to a full year of safety monitoring.

The most common reported TEAEs were akathisia (30.7%), nasopharyngitis (26.6%), nausea (12.1%), and somnolence (12.1%). The results of the current study found lurasidone to have a low incidence of acute EPS and suggest that long-term treatment with lurasidone is associated with a low risk of tardive extrapyramidal symptoms. However, akathisia was notably higher than reported in two previous short-term studies of lurasidone in bipolar depression. In the Loebel et al. study (2014a) akathisia rates were 7.9% and 10.8% for the 20–60 mg and 80–120 mg dosage groups, respectively. In the Kato et al. study (2020) akathisia rates were 13.0% and 23.7% for the 20–60 mg and 80–120 mg dosage groups, respectively. The higher rate of akathisia observed in the current study appears to be attributable to the fact that the placebo-treated subgroup of depressed patients, that entered the current study after completing the Kato et al. study (2020) was switched to an initial dose of 60 mg of lurasidone with no titration. In contrast, patients in the previous short-term studies (Loebel et al. 2014a; Kato et al. 2020) were treated initially with a 20 mg dose for 7 days prior to gradual titration up to their assigned fixed dosage group.

Over the course of 52 weeks of lurasidone treatment, there were no meaningful mean changes (largely decreases) in total cholesterol, LDL cholesterol, blood glucose, haemoglobin A1C, or prolactin. Small mean increases in triglycerides (mean change = 5.4 mg/dL) and HOMA-IR (mean change = 0.37) were observed. By way of comparison, in the Kanba et al. (2019) study of 52 weeks of quetiapine monotherapy for Japanese patients with bipolar depression, the mean change in triglycerides was 11.8 mg/dL. In the Katagiri et al. (2014) study of 42 or 48 weeks of olanzapine treatment in Japanese patients with bipolar depression, the mean increase in triglycerides was 35.1 mg/dL.

Fifty-two weeks of lurasidone treatment was associated with an average gain of 1.0 kg in body weight in the non-depressed group and a reduction of 0.8 kg in the depressed group. The 6 week study of lurasidone monotherapy for bipolar depression in a non-Japanese sample found a mean change in body weight of 0.6 kg for 20–60 mg/day and 0.0 kg for 80–120 mg/day (Loebel et al. 2014a), and the 6 week study of lurasidone (20–120 mg/day) as an adjunct to a mood stabilizer found a mean change in body weight of 0.2 kg. Mean changes in body weight for long-term treatment with olanzapine and quetiapine in Japanese patients with bipolar depression have been reported to be 3.5 kg and 1.02 kg, respectively (Katagiri et al. 2014; Kanba et al. 2019).

The rates of concomitant medication use in this study was high, with 38.2% of patients receiving antiparkinson medications, 41.2% anxiolytics, and 67.3% hypnotics. It is difficult to compare these concomitant medication rates with other long-term bipolar antipsychotic treatment studies in the literature for multiple reasons, including differences between studies in design (eg., open-label vs. double-blind; adjunctive treatment vs. monotherapy), restrictions on concomitant medications in some studies, failure to report concomitant medication use in certain studies, differences in the patient populations of studies (eg., percent of patients with recent manic/hypomanic vs. depressive episode; Japanese patients vs. other countries), and unique aspects of the current study (eg., mood stabilizers were mandatory for the newly recruited patients but not for those entering the 52 week trial after participating in the 6 week prior trial). It is worth noting that the rate of use of antiparkinson medications was twice as high among the depressed group compared to the non-depressed group in the current study (47.9% vs 24.4%). This may suggest that clinicians should particularly attend to Parkinson symptoms for bipolar patients with a recent depressive episode. However, in a previous 24 week study of lurasidone for bipolar depression, only 9.5% of patients receiving adjunctive lurasidone, and 4.4% of those receiving monotherapy lurasidone, received treatment with anticholinergic medication for acute extrapyramidal symptoms.

About half (50.3%) of the sample did not complete the full 52 week treatment period. This discontinuation rate appears to be comparable to discontinuation rates reported in previous studies of atypical antipsychotics for the long-term treatment of bipolar disorder. For example, 52 weeks of treatment with the extended-release formulation quetiapine (monotherapy) was associated with a 43% discontinuation rate (Kanba et al. 2019); 46 weeks of treatment with aripiprazole (in combination with lithium or valproate) was associated with a 48% discontinuation rate (Vieta et al. 2010); and 48 weeks of olanzapine monotherapy was associated with a 57% discontinuation rate (Katagiri et al. 2014). For olanzapine monotherapy, discontinuation rates of 42% have been reported at 18 weeks (Katagiri et al. 2012), and 34% at 24 weeks (Corya et al. 2006), that are consistent with the 50.3% rate of discontinuation reported in the current study.

Reasons for the 50% discontinuation rate in the current study are uncertain. One possible reason is that the patient group switching from placebo in the initial double-blind study was started on 60 mg of lurasidone, which might be considered to be a high dose associated with an increased likelihood of adverse events. However, the overall discontinuation rate was similar in the placebo group and the group of patients continuing on lurasidone; and the discontinuation rate due to an adverse event, was also similar for both groups.

### Effectiveness

Notable in the descriptive analysis of symptom measures for the depressed episode group was the maintenance of improvements that had been achieved during the initial 6 week double-blind study. For the non-depressed episode group who had not participated in an initial 6 week study, substantial improvements in mania symptoms occurred during the first 3 months of lurasidone treatment and these improvements were then maintained out to 52 weeks. Overall, about half of those who began the long-term study met a rigorous definition of clinical stability during the 52 week treatment period. Only 12.5% of those in the depressed episode group and 23.5% in those in the non-depressed episode group experienced a relapse/recurrence of a mood event. Though differences in patient populations and definitions of relapse make comparisons to the literature difficult, these rates compare favourably to estimates of a one-year relapse rate of 75% for bipolar disorder patients receiving placebo and 30–40% for bipolar disorder patients receiving lithium (Fleiss et al. 1978).

## Limitations

Study limitations include most notably that no comparison or control group was included and no inferential statistical tests were conducted. Because there was no control group, the changes over time that were found may be due to the passage of time or other factors besides treatment with lurasidone. Another limitation is that treatment was open-label. Any potential differences between patients with a current/recent depressed versus manic/hypomanic would need to be tested in future hypothesis-testing double-blind studies. A further limitation is that about half of the patients who began the study did not complete the full 52 weeks of treatment. A final limitation is that the inclusion/exclusion criteria ruled out patients with psychiatric comorbidities. The generalizability of the current findings to bipolar patients with additional psychiatric disorders is uncertain.

## Conclusion

In summary, over the course of 52 weeks of lurasidone (20–120 mg/day) treatment with or without a mood stabilizer in Japanese patients with bipolar disorder there were no new safety concerns and no average changes in metabolic parameters were small. Body weight increased on average 1.0 kg in the non-depressed group and decreased on average 0.8 kg in the depressed group. Overall, 49.7% completed 52 weeks of study treatment. Akathisia occurred for 30.7%, and nausea for 12.1%, of patients. Patients with a current/recent depressed episode showed continued improvement in depressive symptoms over the treatment period; patients with a current/recent manic, hypomanic, or mixed episode showed reduction in manic symptoms. These findings support the use of lurasidone as a long-term treatment of bipolar disorder in Japanese patients.

## Supplementary Information

**Additional file 1.** Additional efficacy and safety tables and figures.

## Data Availability

Sumitomo Dainippon Pharma makes individual patient, de-identified data sets and associated clinical documents such as study protocol, statistical analysis plan and clinical study report available upon request via the Clinical Study Data Request site (https://www.clinicalstudydatarequest.com/Study-Sponsors.aspx) within 12 months of posting the study results on clininicaltrials.gov. Access is provided after a research proposal is submitted and has received approval from the Independent Review Panel and after a Data Sharing Agreement is in place. Access is provided for an initial period of 12 months but an extension can be granted, when justified, for up to another 12 months.
