# Supplementary material for: Lurasidone in the long-term treatment of Japanese patients with bipolar I disorder: a 52 week open label study
Source: Int J Bipolar Disord. 2021 Aug 2;9:25. doi: 10.1186/s40345-021-00230-8 (PMC8333182; doi:10.1186/s40345-021-00230-8)
Supplement: Supplementary file 1 — Additional file 1. Additional efficacy and safety tables and figures. [file 40345_2021_230_MOESM1_ESM.docx]

Additional file 1

Supplement to: Teruhiko Higuchi, Tadafumi Kato, Mari Miyajima, Kei Watabe, Takahiro Masuda, Katsuhiko Hagi, Jun Ishigooka.

Lurasidone in The Long-Term Treatment of Japanese Patients with Bipolar I Disorder: A 52-Week Open-Label Study.

Teruhiko Higuchi, et al.

Lurasidone in The Long-Term Treatment of Japanese Patients with Bipolar I Disorder: A 52-Week Open-Label Study.

Additional file 1

**Contents**

[**Supplementary Table S1. Baseline characteristics of patients treated in 52-week open-label study** 3](#_Toc58315012)

[**Supplementary Table S2. Treatment-emergent adverse events (safety population; *N* [%]; incidence ≥ 5%)** 5](#_Toc58315013)

[**Supplementary Table S3. Deaths and other significant adverse events** 6](#_Toc58315014)

[**Supplementary Table S4. Body weight and laboratory parameters: mean (SD) change from long-term baseline to week 52 (observed case analysis)** 7](#_Toc58315015)

[**Supplementary Table S5.** **Efficacy and Safety Measures: mean change (SD) from baseline to LOCF endpoint** 9](#_Toc58315016)

[**Supplementary Table S6. Summary of recurrence/relapse criteria including clinical stability** 10](#_Toc58315017)

[**Supplementary Fig. S1. Time course of mean MADRS total score** 12](#_Toc58315018)

[**Supplementary Fig. S2. Time course of mean MADRS total score by mood stabilizer** 13](#_Toc58315019)

[**A) Lithium** 13](#_Toc58315020)

[**B) VPA** 14](#_Toc58315021)

[**C) Without mood stabilizer** 15](#_Toc58315022)

[**Supplementary Fig. S3. Time course of mean YMRS score** 16](#_Toc58315023)

[**Supplementary Fig. S4. Time course of mean YMRS score by mood stabilizer** 17](#_Toc58315024)

[**A) Lithium** 17](#_Toc58315025)

[**B) VPA** 18](#_Toc58315026)

[**C) Without mood stabilizer** 19](#_Toc58315027)

**Supplementary Table S1. Baseline characteristics of patients treated in 52-week open-label study**

|  | **Most Recent or Current Episode of Bipolar I Disorder** | | | | | | | | |
| --- | --- | --- | --- | --- | --- | --- | --- | --- | --- |
|  | **All Patients**  **(n=199)** | **Depressed episode** | | |  | **Non-Depressed episode** | | | |
|  |  | **Overall (n=117)** | **PBO to LUR**  **(n=41)** | **LUR to LUR**  **(n=76)** |  | **Overall**  **(n=82)** | **Manic**  **(n=43)** | **Mixed**  **(n=12)** | **Hypomanic**  **(n=27)** |
| **Demographics and Clinical Characteristics** | | | | | | | | | |
| **Male, n (%)** | 102 (51.3) | 63 (53.8) | 21 (51.2) | 42 (55.3) |  | 39 (47.6) | 19 (44.2) | 1 (8.3) | 19 (70.4) |
| **Age (years), mean (SD)** | 41.6 (12.0) | 40.0 (10.9) | 39.5 (10.7) | 40.3 (11.1) |  | 43.8 (13.1) | 43.3 (13.6) | 41.5 (10.8) | 45.5 (13.4) |
| **Duration of bipolar I disorder (years),**  **mean (SD)** | 13.5 (11.1) | 13.8 (10.6) | 12.7 (7.6) | 14.4 (11.9) |  | 13.0 (11.7) | 12.9 (12.8) | 14.1 (11.3) | 12.6 (10.6) |
| **With rapid cycling,  n (%)** | 30 (15.1) | 15 (12.8) | 6 (14.6) | 9 (11.8) |  | 15 (18.3) | 5 (11.6) | 4 (33.3) | 6 (22.2) |
| **Efficacy measure** | | | | | | | | | |
| **MADRS, mean (SD)** | 12.4 (10.3) | 16.0 (10.2) | 18.2 (9.9) | 14.8 (10.2) |  | 7.3 (8.0) | 4.6 (4.1) | 21.0 (9.5) | 5.6 (5.8) |
| **CGI-BP-S Overall,**  **mean (SD)** | 2.98 (1.29) | 3.10 (1.25) | 3.39 (1.24) | 2.95 (1.23) |  | 2.80 (1.33) | 2.63 (1.42) | 3.92 (0.90) | 2.59 (1.12) |
| **CGI-BP-S Depression, mean (SD)** | 2.51 (1.39) | 3.03 (1.31) | 3.29 (1.38) | 2.89 (1.26) |  | 1.77 (1.13) | 1.35 (0.57) | 3.67 (1.07) | 1.59 (0.97) |
| **CGI-BP-S Mania,**  **mean (SD)** | 1.77 (1.18) | 1.23 (0.56) | 1.22 (0.53) | 1.24 (0.59) |  | 2.55 (1.39) | 2.51 (1.52) | 3.00 (1.35) | 2.41 (1.19) |
| **SDS, mean (SD)#** | 11.9 (8.7) | 13.7 (9.03) | 15.1 (8.3) | 12.9 (9.4) |  | 9.2 (7.6) | 8.3 (7.6) | 16.7 (5.2) | 7.9 (6.9) |
| **YMRS, mean (SD)** | 4.7 (6.7) | 2.0 (2.8) | 2.3 (3.3) | 1.8 (2.4) |  | 8.7 (8.6) | 9.0 (9.0) | 12.6 (9.5) | 6.4 (6.7) |
| **HAM-A, mean (SD)** | 8.3 (7.2) | 10.6 (7.5) | 11.7 (6.9) | 10.0 (7.8) |  | 5.0 (5.2) | 3.4 (3.4) | 11.7 (5.9) | 4.7 (5.0) |
| **Safety Measure** | | | | | | | | | |
| **DIEPSS (excluding overall severity),**  **mean (SD)** | 0.36 (0.78) | 0.50 (0.87) | 0.39 (0.83) | 0.55 (0.89) |  | 0.16 (0.60) | 0.07 (0.34) | 0.17 (0.39) | 0.30 (0.91) |

# Number of patients: All=181, PBO to LUR=39, LUR to LUR=70, Manic=39, Mixed=9, Hypomanic=24

PBO to LUR = received placebo during prior 6-week trial followed by lurasidone (flexibly dosed 20 to 120 mg/day) in 52-week long-term trial.

LUR to LUR = received lurasidone (flexibly dosed at either 20-60 or 80-120 mg/day) during prior 6-week trial followed by lurasidone (flexibly dosed 20 to 120 mg/day) in 52-week long-term trial.

**Abbreviations:**

CGI-BP-S= Clinical Global Impression Bipolar Version Severity of illness score, DIEPSS=Drug-Induced Extrapyramidal Symptoms Scale, HAM-A= Hamilton Rating Scale for Anxiety total score, LUR=lurasidone, MADRS= Montgomery-Åsberg Depression Rating Scale, PBO=placebo, SD=Standard Deviation, SDS= Sheehan Disability Scale, YMRS= Young Mania Rating Scale

**Supplementary Table S2. Treatment-emergent adverse events (safety population; *N* [%]; incidence ≥ 5%)**

| **Adverse event** | **Most Recent or Current Episode of Bipolar I Disorder** | | | | | | | | |
| --- | --- | --- | --- | --- | --- | --- | --- | --- | --- |
|  | **All (n=199)** | **Depressed episode** | | |  | **Non-Depressed episode** | | | |
|  |  | **Overall**  **(n=117)** | **PBO to LUR**  **(n=41)** | **LUR to LUR**  **(n=76)** |  | **Overall**  **(n=82)** | **Manic**  **(n=43)** | **Mixed**  **(n=12)** | **Hypomanic**  **(n=27)** |
| **At least one adverse event** | 169 (84.9%) | 96 (82.1) | 36 (87.8%) | 60 (78.9%) |  | 73 (89.0%) | 35 (81.4%) | 12 (100.0%) | 26 (96.3%) |
| **Akathisia** | 61 (30.7%) | 40 (34.2%) | 17 (41.5%) | 23 (30.3%) |  | 21 (25.6%) | 9 (20.9%) | 4 (33.3%) | 8 (29.6%) |
| **Nasopharyngitis** | 53 (26.6%) | 24 (20.5%) | 10 (24.4%) | 14 (18.4%) |  | 29 (35.4%) | 11 (25.6%) | 4 (33.3%) | 14 (51.9%) |
| **Nausea** | 24 (12.1%) | 12 (10.3%) | 6 (14.6%) | 6 (7.9%) |  | 12 (14.6%) | 7 (16.3%) | 3 (25.0%) | 2 (7.4%) |
| **Somnolence** | 24 (12.1%) | 10 (8.5%) | 5 (12.2%) | 5 (6.6%) |  | 14 (17.1%) | 9 (20.9%) | 1 (8.3%) | 4 (14.8%) |
| **Weight increased** | 17 (8.5%) | 8 (6.8%) | 3 (7.3%) | 5 (6.6%) |  | 9 (11.0%) | 4 (9.3%) | 2 (16.7%) | 3 (11.1%) |
| **Headache** | 16 (8.0%) | 7 (6.0%) | 4 (9.8%) | 3 (3.9%) |  | 9 (11.0%) | 5 (11.6%) | 2 (16.7%) | 2 (7.4%) |
| **Parkinsonism** | 15 (7.5%) | 10 (8.5%) | 4 (9.8%) | 6 (7.9%) |  | 5 (6.1%) | 2 (4.7%) | 1 (8.3%) | 2 (7.4%) |
| **Disease progression** | 13 (6.5%) | 9 (7.7%) | 3 (7.3%) | 6 (7.9%) |  | 4 (4.9%) | 2 (4.7%) | 2 (16.7%) | 0 |
| **Vomiting** | 13 (6.5%) | 8 (6.8%) | 3 (7.3%) | 5 (6.6%) |  | 5 (6.1%) | 3 (7.0%) | 1 (8.3%) | 1 (3.7%) |
| **Diarrhoea** | 10 (5.0%) | 4 (3.4%) | 2 (4.9%) | 2 (2.6%) |  | 6 (7.3%) | 1 (2.3%) | 0 | 5 (18.5%) |
| **Dystonia** | 10 (5.0%) | 5 (4.3%) | 3 (7.3%) | 2 (2.6%) |  | 5 (6.1%) | 3 (7.0%) | 1 (8.3%) | 1 (3.7%) |

PBO to LUR = received placebo during prior 6-week trial followed by lurasidone (flexibly dosed 20 to 120 mg/day) in 52-week long-term trial.

LUR to LUR = received lurasidone (flexibly dosed at either 20-60 or 80-120 mg/day) during prior 6-week trial followed by lurasidone (flexibly dosed 20 to 120 mg/day) in 52-week long-term trial.

**Abbreviations:**

LUR=lurasidone, PBO=placebo

**Supplementary Table S3. Deaths and other significant adverse events**

| **Adverse event** | **Most Recent or Current Episode of Bipolar I Disorder** | | | | | | | | |
| --- | --- | --- | --- | --- | --- | --- | --- | --- | --- |
|  | **All (n=199)** | **Depressed episode** | | |  | **Non-Depressed episode** | | | |
|  |  | **Overall**  **(n=117)** | **PBO to LUR**  **(n=41)** | **LUR to LUR**  **(n=76)** |  | **Overall**  **(n=82)** | **Manic**  **(n=43)** | **Mixed**  **(n=12)** | **Hypomanic**  **(n=27)** |
| **Death** | 0 | 0 | 0 | 0 |  | 0 | 0 | 0 | 0 |
| **Suicidal ideation** | 4 (2.0%) | 2 (1.7%) | 0 | 2 (2.6%) |  | 2 (2.4%) | 1 (2.3%) | 1 (8.3) | 0 |
| **Suicide attempt** | 2 (1.0%) | 2 (1.7%) | 1 (2.4%) | 1 (1.3%) |  | 0 | 0 | 0 | 0 |
| **ECG QT prolonged ^a)^** | 0 | 0 | 0 | 0 |  | 0 | 0 | 0 | 0 |
| **Treatment emergent mania ^b)^** | 20 (10.1%) | 1 (0.9%) | 1 (2.4%) | 0 |  | 19 (23.2%) | 13 (30.2%) | 2 (16.7%) | 4 (14.8%) |
| **≥7% weight increase^†^** | 35 (17.7%) | 18 (15.4%) | 7 (17.1%) | 11 (14.5%) |  | 17 (21.0%) | 10 (23.8%) | 3 (25.0%) | 4 (14.8%) |
| **≥7% weight decrease^†^** | 29 (14.6%) | 19 (16.2%) | 6 (14.6%) | 13 (17.1%) |  | 10 (12.3%) | 4 (9.5%) | 3 (25.0%) | 3 (11.1%) |

† Number of patients: All=198, PBO to LUR=41, LUR to LUR=76, Manic=42, Mixed=12, Hypomanic=27

1. Fridericia’s corrected QT interval of greater than 500ms or an increase of at least 60ms at any time during the study.
2. Treatment-emergent mania is defined as a YMRS total score of ≥16 at any 2 consecutive post-long-term-baseline visits or at the final assessment, or patients of mania and hypomania as an adverse event.

**Supplementary Table S4. Body weight and laboratory parameters: mean (SD) change from long-term baseline to week 52 (observed case analysis)**

| **Parameter** | **Most Recent or Current Episode of Bipolar I Disorder** | | | | | | | | |
| --- | --- | --- | --- | --- | --- | --- | --- | --- | --- |
|  | **All** | **Depressed episode** | | |  | **Non-Depressed episode** | | | |
|  |  | **Overall** | **PBO to LUR** | **LUR to LUR** |  | **Overall** | **Manic** | **Mixed** | **Hypomanic** |
| **Body weight, kg**  **Mean change**  **Median change** | n=99  0.0 (5.6)  0.1 | n=52  -0.8 (5.6)  0.1 | n=20  -0.3 (5.6)  0.6 | n=32  -1.1 (5.7)  -0.4 |  | n=47   1. (5.5)   0.1 | n=24  1.6 (5.4)  1.1 | n=7  -0.4 (7.0)  1.9 | n=16  0.6 (5.3)  -0.6 |
| **Triglycerides (mg/dl) -fasting**  **Mean change**  **Median change** | n=99  5.4 (59.1)  6.0 | n=52  10.0 (64.9)  6.5 | n=20  6.9 (49.5)  8.0 | n=32  11.9 (73.7)  5.0 |  | n=47  0.3 (52.0)  5.0 | n=24  -7.0 (41.8)  -1.5 | n=7  -1.9 (25.5)  1.0 | n=16  12.1 (71.5)  10.0 |
| **Total cholesterol (mg/dl) -fasting**  **Mean change**  **Median change** | n=99  -3.1 (25.3)  -1.0 | n=52  -2.5 (26.8)  -3.5 | n=20  0.5 (26.4)  -3.0 | n=32  -4.4 (27.3)  -3.5 |  | n=47  -3.8 (23.8)  2.0 | n=24  0.8 (21.2)  8.0 | n=7  -7.0 (17.0)  -1.0 | n=16  -9.2 (29.4)  -1.0 |
| **LDL cholesterol (mg/dl) -fasting**  **Mean change**  **Median change** | n=97  -1.5 (21.1)  1.0 | n=50  -2.5 (23.3)  1.5 | n=20  -0.6 (21.8)  2.0 | n=30  -3.7 (24.6)  0.0 |  | n=47  -0.4 (18.6)  1.0 | n=24  3.0 (17.0) 5.0 | n=7  -4.7 (28.4)  2.0 | n=16  -3.8 (16.1)  -3.0 |
| **HDL cholesterol (mg/dl) -fasting**  **Mean change**  **Median change** | n=99  -1.8 (10.7)  -2.0 | n=52  -0.7 (9.9)  -1.5 | n=20  -0.3 (8.5)  -1.5 | n=32  -1.0 (10.8)  -1.5 |  | n=47  -3.0 (11.5)  -2.0 | n=24  -0.8 (8.0)  0.0 | n=7  -1.9 (15.6)  -4.0 | n=16  -6.9 (13.6)  -3.0 |
| **Blood glucose (mg/dl) -fasting**  **Mean change**  **Median change** | n=99  0.8 (10.9)  2.0 | n=52  0.8 (12.4)  2.0 | n=20  0.0 (11.1)  2.5 | n=32  1.3 (13.3)  2.0 |  | n=47  0.8 (9.1)  1.0 | n=24  1.2 (8.17)  1.5 | n=7  1.3 (14.0)  2.0 | n=16   1. (8.4)   -3.0 |
| **Insulin (mU/liter) -fasting** | n=98  1.39 (9.15)  1.40 | n=51  1.21 (10.95)  1.30 | n=20  -0.23 (8.39)  0.35 | n=31  2.14 (12.37)  1.60 |  | n=47  1.59 (6.79)  1.50 | n=24  0.78 (5.79)  1.10 | n=7  0.71 (6.47)  1.80 | n=16  3.19 (8.31)  2.15 |
| **HOMA-IR -fasting**  **Mean change**  **Median change** | n=98  0.37 (2.76)  0.33 | n=51  0.34 (3.46)  0.30 | n=20  -0.20 (2.72)  -0.04 | n=31  0.69 (3.87) 0.46 |  | n=47  0.40 (1.73)  0.35 | n=24  0.20 (1.60)  0.32 | n=7  0.20 (1.46)  0.45 | n=16  0.78 (2.05)  0.47 |
| **Hemoglobin A1C (%)**  **Mean change**  **Median change** | n=99  0.00 (0.29)  0.00 | n=52  -0.01 (0.35)  0.00 | n=20  0.07 (0.37)  0.10 | n=32  -0.05 (0.34)  0.00 |  | n=47  0.01 (0.20)  0.00 | n=24  0.00 (0.24)  0.00 | n=7  -0.03 (0.08)  0.00 | n=16  0.04 (0.16)  0.00 |
| **Prolactin, (ng/ml), Overall**  **Mean change**  **Median change** | n=98  -1.7 (17.6)  -0.5 | n=51  -5.5 (18.7)  -1.3 | n=20  1.1 (6.8)  -0.4 | n=31  -9.8 (22.5)  -3.5 |  | n=47  2.3 (15.5)  1.3 | n=24  -0.4 (12.2)  1.6 | n=7  9.1 (31.7)  -2.5 | n=16  3.5 (8.2)  1.4 |
| **Prolactin, (ng/ml), Male**  **Mean change**  **Median change** | n=44  -0.3 (9.2)  -0.3 | n=24  0.3 (7.5)  -0.5 | n=11  1.8 (6.4)  0.2 | n=13  -1.0 (8.3)  -1.2 |  | n=20  -0.9 (11.1)  0.3 | n=9  -4.5 (14.2)  0.9 | n=0 | n=11  2.1 (7.2)  -0.6 |
| **Prolactin, (ng/ml), Female**  **Mean change**  **Median change** | n=54  -3.0 (22.2)  -0.6 | n=27  -10.6 (23.8)  -2.6 | n=9  0.3 (7.6)  -0.7 | n=18  -16.1 (27.3)  -5.7 |  | n=27  4.7 (17.9)  3.7 | n=15  2.0 (10.7)  3.7 | n=7  9.1 (31.7)  -2.5 | n=5  6.7 (10.2)  5.6 |

PBO to LUR = received placebo during prior 6-week trial followed by lurasidone (flexibly dosed 20 to 120 mg/day) in 52-week long-term trial.

LUR to LUR = received lurasidone (flexibly dosed at either 20-60 or 80-120 mg/day) during prior 6-week trial followed by lurasidone (flexibly dosed 20 to 120 mg/day) in 52-week long-term trial.

**Abbreviations:**

LUR=lurasidone, PBO=placebo

**Supplementary Table S5.** **Efficacy and Safety Measures: mean change (SD) from baseline to LOCF endpoint**

|  | **Most Recent or Current Episode of Bipolar I Disorder** | | | | | | | | |
| --- | --- | --- | --- | --- | --- | --- | --- | --- | --- |
|  | **All (n=199)** | **Depressed episode** | | |  | **Non-Depressed episode** | | | |
|  |  | **Overall**  **(n=117)** | **PBO to LUR**  **(n=41)** | **LUR to LUR**  **(n=76)** |  | **Overall**  **(n=82)** | **Manic**  **(n=43)** | **Mixed**  **(n=12)** | **Hypomanic**  **(n=27)** |
| **Measure** | **Mean (SD)** | **Mean (SD)** | **Mean (SD)** | **Mean (SD)** |  | **Mean (SD)** | **Mean (SD)** | **Mean (SD)** | **Mean (SD)** |
| **Efficacy** |  |  |  |  |  |  |  |  |  |
| **MADRS** | 1.1 (12.6) | 0.2 (13.6) | -2.0 (14.7) | 1.4 (12.9) |  | 2.5 (10.9) | 4.1 (10.6) | -5.8 (12.5) | 3.7 (9.1) |
| **YMRS** | -2.0 (6.7) | -0.1 (3.5) | -0.6 (3.4) | 0.2 (3.6) |  | -4.8 (9.0) | -3.5 (10.1) | -9.7 (9.1) | -4.7 (6.1) |
| **CGI-BP-S Overall** | -0.09 (1.57) | -0.06 (1.51) | -0.39 (1.60) | 0.12 (1.43) |  | -0.14 (1.66) | 0.14 (1.86) | -0.92 (1.56) | -0.22 (1.25) |
| **CGI-BP-S Depression** | 0.14 (1.58) | -0.03 (1.61) | -0.32 (1.64) | 0.13 (1.58) |  | 0.38 (1.53) | 0.57 (1.40) | -0.75 (1.55) | 0.59 (1.55) |
| **CGI-BP-S Mania** | -0.36 (1.13) | -0.01 (0.66) | -0.07 (0.52) | 0.03 (0.73) |  | -0.86 (1.43) | -0.48 (1.53) | -1.92 (1.51) | -1.00 (0.92) |
| **HAM-A†** | 0.0 (7.8) | -0.6 (8.7) | -1.1 (8.5) | -0.3 (8.9) |  | 0.9 (6.2) | 2.0 (6.1) | -2.8 (7.8) | 0.8 (5.1) |
| **SDS#** | -0.1 (9.9) | 0.4 (10.0) | -0.4 (10.7) | 0.8 (9.7) |  | -0.9 (9.9) | -0.1 (10.4) | -8.7 (8.3) | 0.9 (8.4) |
| **Safety** |  |  |  |  |  |  |  |  |  |
| **DIEPSS Total Score¶**  **(excluding overall severity)** | 0.25 (1.20) | 0.20 (1.23) | 0.59 (1.20) | -0.01 (1.19) |  | 0.33 (1.16) | 0.40 (1.38) | 0.17 (0.72) | 0.30 (0.95) |

† Number of patients: All=197, PBO to LUR=41, LUR to LUR=75, Manic=42, Mixed=12, Hypomanic=27

# Number of patients: All=177, PBO to LUR=38, LUR to LUR=69, Manic=37, Mixed=9, Hypomanic=24

¶ Number of patients: All=198, PBO to LUR=41, LUR to LUR=76, Manic=42, Mixed=12, Hypomanic=27

PBO to LUR = received placebo during prior 6-week trial followed by lurasidone (flexibly dosed 20 to 120 mg/day) in 52-week long-term trial.

LUR to LUR = received lurasidone (flexibly dosed at either 20-60 or 80-120 mg/day) during prior 6-week trial followed by lurasidone (flexibly dosed 20 to 120 mg/day) in 52-week long-term trial.

**Abbreviations:**

CGI-BP-S= Clinical Global Impression Bipolar Version Severity of illness score, DIEPSS=Drug-Induced Extrapyramidal Symptoms Scale, HAM-A= Hamilton Rating Scale for Anxiety total score, LUR=lurasidone, MADRS= Montgomery-Åsberg Depression Rating Scale, PBO=placebo, SD=Standard Deviation, SDS= Sheehan Disability Scale, YMRS= Young Mania Rating Scale

**Supplementary Table S6. Summary of recurrence/relapse criteria including clinical stability**

| **Recurrence/Relapse Criteria** | **Most Recent or Current Episode of Bipolar I Disorder** | | | | | | | | |
| --- | --- | --- | --- | --- | --- | --- | --- | --- | --- |
|  | **All (n=199)** | **Depressed episode** | | |  | **Non-Depressed episode** | | | |
|  |  | **Overall**  **(n=117)** | **PBO to LUR**  **(n=41)** | **LUR to LUR**  **(n=76)** |  | **Overall**  **(n=82)** | **Manic**  **(n=43)** | **Mixed**  **(n=12)** | **Hypomanic**  **(n=27)** |
| **Clinical stability ^†^** | 103 (51.8%) | 52 (44.4%) | 21 (51.2%) | 31 (40.8%) |  | 51 (62.2%) | 30 (69.8%) | 5 (41.7%) | 16 (59.3%) |
| **Duration of stabilization Mean days (SD)** | 141.3 (79.94) | 160.6 (92.41) | 150.4 (98.28) | 167.5 (89.21) |  | 121.7 (59.58) | 112.6 (51.53) | 201.2 (89.04) | 114.0 (47.44) |
| **Met any Recurrence/Relapse Criteria ^¶^** | 18 (17.5%) | 6 (11.5%) | 3 (14.3%) | 3 (9.7%) |  | 12 (23.5%) | 9 (30.0%) | 1 (20.0%) | 2 (12.5%) |
| 1. **Fulfilled DSM-IV-TR criteria for depressed, manic, mixed or hypomanic episode** | 7 (6.8%) | 4 (7.7%) | 3 (14.3%) | 1 (3.2%) |  | 3 (5.9%) | 1 (3.3%) | 1 (20.0%) | 1 (6.3%) |
| **Depressed** | 6 (5.8%) | 4 (7.7%) | 3 (14.3%) | 1 (3.2%) |  | 2 (3.9%) | 1 (3.3%) | 0 | 1 (6.3%) |
| **Manic** | 0 | 0 | 0 | 0 |  | 0 | 0 | 0 | 0 |
| **Mixed** | 1 (1.0%) | 0 | 0 | 0 |  | 1 (2.0%) | 0 | 0 | 0 |
| **Hypomanic** | 0 | 0 | 0 | 0 |  | 0 | 0 | 0 | 0 |
| 1. **Required treatment intervention for depressed, manic, mixed or hypomanic episode** | 2 (1.9%) | 1 (1.9%) | 0 | 1 (3.2%) |  | 1 (2.0%) | 1 (3.3%) | 0 | 0 |
| 1. **Psychiatric hospitalization for any bipolar mood episode** | 0 | 0 | 0 | 0 |  | 0 | 0 | 0 | 0 |
| 1. **YMRS or MADRS total score ≥18 or CGI-BP-S (overall, depression, mania) ≥4 at two consecutive assessments no more than 10 days apart** | 9 (8.7%) | 2 (3.8%) | 0 | 2 (6.5%) |  | 7 (13.7%) | 6 (20.0%) | 0 | 1 (6.3%) |
| 1. **Discontinuation from the study because of a mood event (as determined by the Investigator)** | 1 (1.0%) | 0 | 0 | 0 |  | 1 (2.0%) | 1 (3.3%) | 0 | 0 |

^†^ Total score of ≤12 on YMRS or MADRS over at least 12 weeks, with the allowance of 2 excursions (YMRS and/or MADRS total scores up to 13 or 14, respectively) except during at least 4 weeks before achieving clinical stability.

^¶^ Percentage in clinically stabilized patients

**Abbreviations:**

CGI-BP-S= Clinical Global Impression Bipolar Version Severity of illness score, DSM-IV-TR= Diagnostic and Statistical Manual of Mental Disorders, fourth edition, Text Revision, MADRS=Montgomery-Åsberg Depression Rating Scale, YMRS=Young Mania Rating Scale

**Supplementary Fig. S1. Time course of mean MADRS total score**

PBO to LUR = received placebo during prior 6-week trial followed by lurasidone (flexibly dosed 20 to 120 mg/day) in 52-week long-term trial.

LUR to LUR = received lurasidone (flexibly dosed at either 20-60 or 80-120 mg/day) during prior 6-week trial followed by lurasidone (flexibly dosed 20 to 120 mg/day) in 52-week long-term trial.

NR to LUR = newly recruited Japanese subjects whose most recent or current episode was mania, hypomania, or mixed and received lurasidone (flexibly dosed 20 to 120 mg/day) in 52-week long-term trial.

**Abbreviations:**

LOCF=last observation carried forward, LT=long-term, LUR=lurasidone, MADRS=Montgomery-Åsberg Depression Rating Scale, NR=newly recruited, PBO=placebo, YMRS=Young Mania Rating Scale

**Supplementary Fig. S2. Time course of mean MADRS total score by mood stabilizer**

**A) Lithium**

PBO to LUR = received placebo during prior 6-week trial followed by lurasidone (flexibly dosed 20 to 120 mg/day) in 52-week long-term trial.

LUR to LUR = received lurasidone (flexibly dosed at either 20-60 or 80-120 mg/day) during prior 6-week trial followed by lurasidone (flexibly dosed 20 to 120 mg/day) in 52-week long-term trial.

NR to LUR = newly recruited Japanese subjects whose most recent or current episode was mania, hypomania, or mixed and received lurasidone (flexibly dosed 20 to 120 mg/day) in 52-week long-term trial.

**Abbreviations:**

LOCF=last observation carried forward, LT=long-term, LUR=lurasidone, MADRS=Montgomery-Åsberg Depression Rating Scale, NR=newly recruited, PBO=placebo

**B) VPA**

PBO to LUR = received placebo during prior 6-week trial followed by lurasidone (flexibly dosed 20 to 120 mg/day) in 52-week long-term trial.

LUR to LUR = received lurasidone (flexibly dosed at either 20-60 or 80-120 mg/day) during prior 6-week trial followed by lurasidone (flexibly dosed 20 to 120 mg/day) in 52-week long-term trial.

NR to LUR = newly recruited Japanese subjects whose most recent or current episode was mania, hypomania, or mixed and received lurasidone (flexibly dosed 20 to 120 mg/day) in 52-week long-term trial.

**Abbreviations:**

LOCF=last observation carried forward, LT=long-term, LUR=lurasidone, MADRS=Montgomery-Åsberg Depression Rating Scale, NR=newly recruited, PBO=placebo, VPA=valproate divalproex

**C) Without mood stabilizer**

PBO to LUR = received placebo during prior 6-week trial followed by lurasidone (flexibly dosed 20 to 120 mg/day) in 52-week long-term trial.

LUR to LUR = received lurasidone (flexibly dosed at either 20-60 or 80-120 mg/day) during prior 6-week trial followed by lurasidone (flexibly dosed 20 to 120 mg/day) in 52-week long-term trial.

NR to LUR = newly recruited Japanese subjects whose most recent or current episode was mania, hypomania, or mixed and received lurasidone (flexibly dosed 20 to 120 mg/day) in 52-week long-term trial.

**Abbreviations:**

LOCF=last observation carried forward, LT=long-term, LUR=lurasidone, MADRS=Montgomery-Åsberg Depression Rating Scale, NR=newly recruited, PBO=placebo

**Supplementary Fig. S3. Time course of mean YMRS score**

PBO to LUR = received placebo during prior 6-week trial followed by lurasidone (flexibly dosed 20 to 120 mg/day) in 52-week long-term trial.

LUR to LUR = received lurasidone (flexibly dosed at either 20-60 or 80-120 mg/day) during prior 6-week trial followed by lurasidone (flexibly dosed 20 to 120 mg/day) in 52-week long-term trial.

NR to LUR = newly recruited Japanese subjects whose most recent or current episode was mania, hypomania, or mixed and received lurasidone (flexibly dosed 20 to 120 mg/day) in 52-week long-term trial.

**Abbreviations:**

LOCF=last observation carried forward, LT=long-term, LUR=lurasidone, NR=newly recruited, PBO=placebo, YMRS=Young Mania Rating Scale

**Supplementary Fig. S4. Time course of mean YMRS score by mood stabilizer**

**A) Lithium**

PBO to LUR = received placebo during prior 6-week trial followed by lurasidone (flexibly dosed 20 to 120 mg/day) in 52-week long-term trial.

LUR to LUR = received lurasidone (flexibly dosed at either 20-60 or 80-120 mg/day) during prior 6-week trial followed by lurasidone (flexibly dosed 20 to 120 mg/day) in 52-week long-term trial.

NR to LUR = newly recruited Japanese subjects whose most recent or current episode was mania, hypomania, or mixed and received lurasidone (flexibly dosed 20 to 120 mg/day) in 52-week long-term trial.

**Abbreviations:**

LOCF=last observation carried forward, LT=long-term, LUR=lurasidone, NR=newly recruited, PBO=placebo, YMRS=Young Mania Rating Scale

**B) VPA**

PBO to LUR = received placebo during prior 6-week trial followed by lurasidone (flexibly dosed 20 to 120 mg/day) in 52-week long-term trial.

LUR to LUR = received lurasidone (flexibly dosed at either 20-60 or 80-120 mg/day) during prior 6-week trial followed by lurasidone (flexibly dosed 20 to 120 mg/day) in 52-week long-term trial.

NR to LUR = newly recruited Japanese subjects whose most recent or current episode was mania, hypomania, or mixed and received lurasidone (flexibly dosed 20 to 120 mg/day) in 52-week long-term trial.

**Abbreviations:**

LOCF=last observation carried forward, LT=long-term, LUR=lurasidone, NR=newly recruited, PBO=placebo, YMRS=Young Mania Rating Scale, VPA=valproate divalproex

**C) Without mood stabilizer**

PBO to LUR = received placebo during prior 6-week trial followed by lurasidone (flexibly dosed 20 to 120 mg/day) in 52-week long-term trial.

LUR to LUR = received lurasidone (flexibly dosed at either 20-60 or 80-120 mg/day) during prior 6-week trial followed by lurasidone (flexibly dosed 20 to 120 mg/day) in 52-week long-term trial.

NR to LUR = newly recruited Japanese subjects whose most recent or current episode was mania, hypomania, or mixed and received lurasidone (flexibly dosed 20 to 120 mg/day) in 52-week long-term trial.

**Abbreviations:**

LOCF=last observation carried forward, LT=long-term, LUR=lurasidone, NR=newly recruited, PBO=placebo, YMRS=Young Mania Rating Scale,
